# A Floral Fragrance, Methyl Benzoate, is An Efficient Green Pesticide

**DOI:** 10.1038/srep42168

**Published:** 2017-02-09

**Authors:** Yan Feng, Aijun Zhang

**Affiliations:** 1 US Department of Agriculture, Agricultural Research Service, Invasive Insect Biocontrol and Behavior Laboratory, Beltsville, Maryland, USA

## Abstract

Over-reliance on synthetic pesticides in insect pest control has caused widespread public and scientific concerns for human health and the environment, especially since many insect pests have already developed resistances to conventional pesticides and *Bt* products. For this reason, there is a considerable interest in development of alternative control methods for insect pest management. Based on laboratory studies, we report that methyl benzoate (MB), a naturally-occurring compound in many plants, may possess toxicity against various stages of a variety of insect pests, including the brown marmorated stinkbug, *Halyomorpha halys*, diamondback moth, *Plutella xylostella*, and tobacco hornworm, *Manduca sexta,* as well as the spotted wing drosophila, *Drosophila suzukii*. Based on our laboratory toxicity data, MB was at least 5 to 20 times more toxic than the conventional pyrethroid (*β*-cyfluthrin), sulfur & pyrethrin mixture, and some organic commercial products available on the market against *H. halys, P. xylostella*, and *M. sexta,* eggs. Because MB is considered an environment-friendly, it has great potential to be used as an alternative tool to synthetic pesticide for insect pest management in crop production, thereby, reducing threats to natural ecosystems and human health caused by over-application of conventional synthetic pesticides.

Several studies have suggested that the increasing global food demand for direct human consumption as result of world population growth, poses unique and huge challenges for the sustainability of crop production^1^,^2^,^3^. Although about 99% of agricultural crop pests, worldwide, are control by natural enemies^4^, crop production can still be severely reduced (in the range of 25–50%) by insects, weeds, and pathogens^5^. Application of synthetic pesticides in pest control has been shown to provide significant economic benefits, allowing farmers to reduce human labor costs in crop production, and made it possible to produce a great volume of food for global consumers^5^. However, this has not been all positive as many insect pests have developed resistances to major classes of synthetic insecticides, and serious damage to human health, agriculture, and natural ecosystems has been attributed to the continuing application of synthetic pesticides. In addition, because insecticides and herbicides are sprayed or spread across entire agricultural fields, it has been estimated that over 98% of sprayed insecticides and 95% of herbicides reach destinations other than their target species, often resulting in harm to non-target wildlife and the environment^6^. Recent estimates have even suggested that pesticides are responsible for more than 20,000 human fatalities, yearly, primarily in developing countries^7^. Thus, there is an urgent need to curtail pesticide use and reduce the human and environmental impacts of synthetic pesticides.

Botanical pesticides have emerged as attractive alternatives to synthetic pesticides presumable because botanicals are perceived as less threatening to human health and the environment^8^,^9^,^10^. A number of botanical pesticides, based on pyrethrum and neem, have been successfully commercialized; however their use in agricultural and veterinary pest control commands only about 1% of the global pesticide market^11^. Therefore, there is still significant opportunity for development of botanical pesticides as environmental-friendly tools in pest management.

Recent investigations have indicated that a volatile organic compound (VOC), methyl benzoate (MB), from the fermented apple juice, exhibits significant repellent activity against an invasive fruit fly, *Drosophila suzukii* Matsumura, also known as spotted wing drosophila (Zhang *et al*. unpublished data). As part of an effort aimed at development of green pesticides based on plant origin, the potential acute toxicity and sublethal effect of MB against *D. suzukii* have been investigated by comparing MB with some monoterpenes, including α-terpinene^12^, γ-terpinene^13^, α-terpineol^11^, α-pinene^14^, and 1,8-cineole^15^ (considered ‘minimum risk pesticides’). The toxicities of these latter compounds have been well demonstrated against several different insect pests. Among all the compounds tested, MB was found to be the most toxic for *D. suzukii*. Since pesticides are usually active against a broad spectrum of pests, MB was also tested against several other pest species, including brown marmorated stinkbug *Halyomorpha halys*, diamondback moth *Plutella xylostella*, and tobacco hornworm *Manduca sexta,* for both acute toxic efficacy and/or sublethal effect. Our results indicated that MB not only effectively prevented egg hatch and inhibited nymph and/or larvae developments of *H. halys, P. xylostella*, and *M. sexta* with contact ovicidal effect, but also had contact insecticidal activity against *H. halys* nymphs.

## Results

### Toxicity of MB to *D. suzukii*

**Figure 1:**
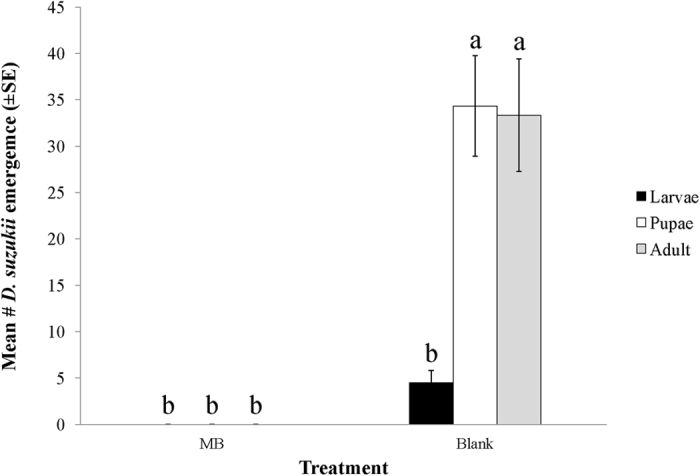
Impact of MB on emergence of *D. suzukii* from pre-infested blueberries (100 berries pre-infested with 100 mixed-adult for 4 days/treatment, 50 berries were then dipped with 1% MB solution and water respectively. Assessment was conducted after 10 days incubation at room temperature. Means flowed by the different letters are significantly different at α = 0.05 (*N* = 6, *F* = 25.472; df = 5,30, *p* < 0.0001).

MB, at a concentration of 1%, exhibited potent toxicity against *D. suzukii* when blueberries were exposed to *D. suzukii* four days prior to treatment (“pre-infested”). Exposure to MB resulted in 100% mortality as no larvae and pupae had developed nor adult flies emerged after 12 days incubation at room temperature (Fig. 1) (*N* = 6, *F* = 25.472; df = 5,30, *p* < 0.0001).

### Comparison of MB to ‘minimum risk’ pesticides against *D. suzukii*

**Figure 2:**
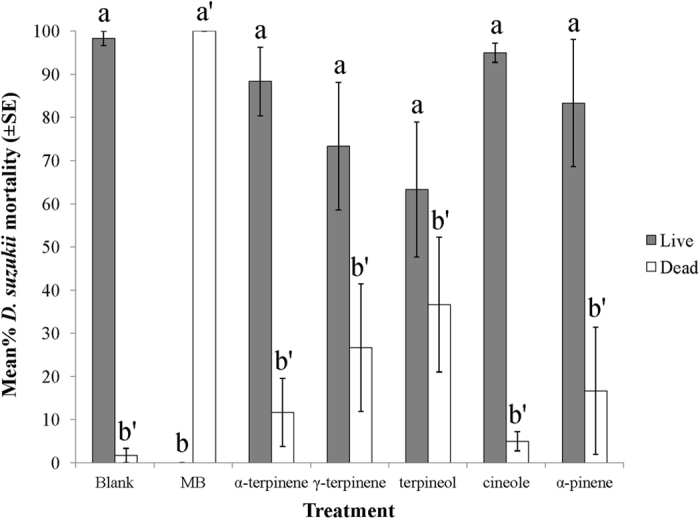
Impact of different VOCs against adult *D. suzukii* (60 flies/treatment). Mortality assessed after 48 hrs exposure to pre-treated blueberries (60 berries/treatment, 1% solution dipping). Means flowed by the different letters and superscripts are significantly different at α = 0.05 (*N* = 6, *F* = 10.691; df = 6,35, *p* < 0.0001).

**Figure 3:**
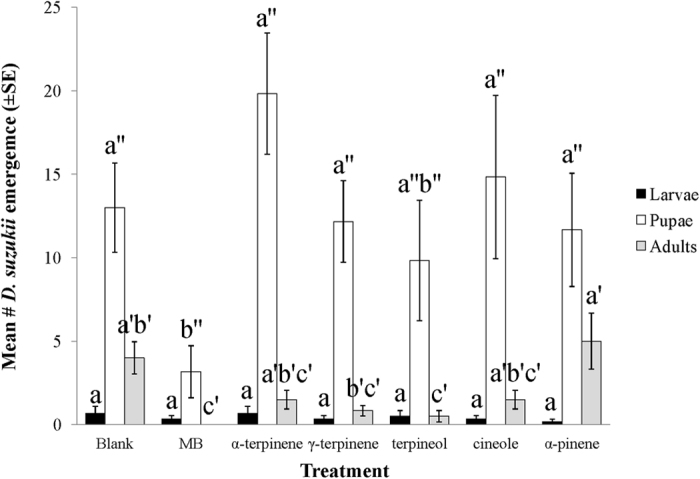
Impact of different VOCs on subsequent *D. suzukii* infestation and development on pre-treated blueberries. Numbers assessed after 10 days incubation at room temperature. Means flowed by the different letters and superscripts are significantly different at α = 0.05 (Log transformed; *N* = 6, df = 6,35; for larvae, *F* = 0.248, *p* > 0.05; for pupae, *F* = 3.586, *p* < 0.01; for adult, *F* = 4.843, *p* < 0.01).

Of all the compounds tested, MB exhibited the most toxicity against *D. suzukii* (Fig. 2). MB exhibited complete mortality and no adult flies survived after two days exposure to pre-treated blueberries (*N* = 6, *F* = 10.691; df = 6,35, *p* < 0.0001). All other essential oils (‘minimum risk pesticides’) tested did not show significant toxicity, when compared to the control. Following further incubation at ambient (room) temperature for 10 days, no adults emerged and significantly fewer pupae developed from MB-treated berries when compared to the blank control or other essential oil treatments (Fig. 3). (*N* = 6, df = 6,35; for adult, *F* = 4.843, *p* < 0.01; for pupae, *F* = 3.586, *p* < 0.01), suggesting that MB also possessed an oviposition deterrent property.

**Figure 4:**
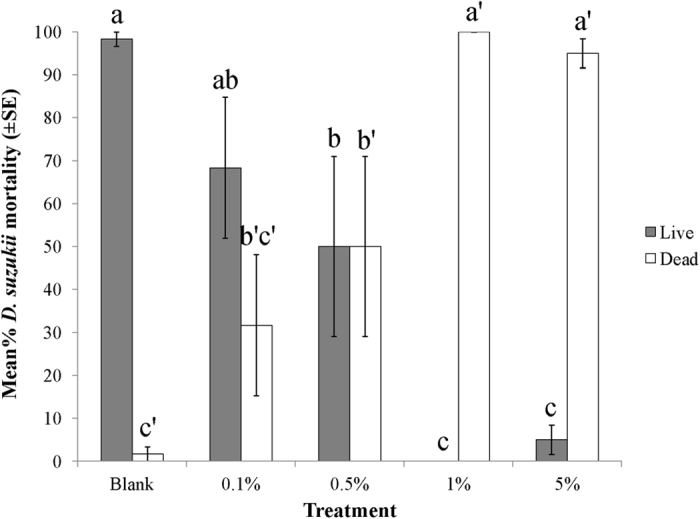
Dose response of MB against adult *D. suzukii* (60 flies/treatment). Mortality assessed after 48 hrs exposure to pre-treated blueberries (60 berries/treatment). Means flowed by the different letters and superscripts are significantly different at α = 0.05 (*N* = 6, *F* = 12.151; df = 4,25, *p* < 0.0001).

**Figure 5:**
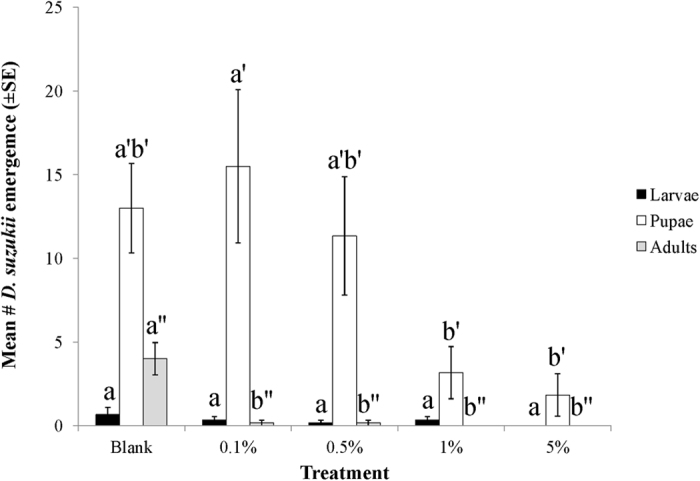
Impact of MB with different doses on subsequent *D. suzukii* infestation and development on pre-treated blueberries. Numbers assessed after 10 days incubation at room temperature. Means flowed by the different letters and superscripts are significantly different at α = 0.05 (Log transformed; *N* = 6, df = 4,25; for larvae, *F* = 1.038, *p* > 0.05; for pupae, *F* = 5.982, *p* < 0.01; for adult, *F* = 27.981, *p *< 0.001).

The toxicity of MB is concentration dependent. After two days exposure to pre-treated blueberries, MB exhibited potent activity against adult *D. suzukii* at 1% and 5% concentrations. Little activity at 0.5% and no significant activity at 0.1% concentrations were observed (Fig. 4). (*N* = 6, *F* = 12.151; df = 4,25, *p* < 0.0001). Following further incubation at room temperature for 10 days, no adults emerged and significantly fewer pupae developed from 1% and 5% MB treated berries comparing to the blank control (Fig. 5). (*N* = 6, df = 6,35; for adult, *F* = 27.981, *p* < 0.001; for pupae, *F* = 5.982, *p* < 0.01; for larvae, *F* = 0.458, *p* > 0.05).

### Toxicity of MB to *H. halys* nymphs

MB also showed contact nymphicidal effect against *H. halys* nymphs. Of the five different stages tested, MB exhibited LC_50_ values from 1.01 to 2.39 μL/vial (Table 1).

### Ovicidal toxicity of MB and commercial pesticides

The ovicidal action of MB was compared to several commercially available organic pest control products (Table 2). The evaluations were conducted by measuring hatchability in direct spray bioassay on three species of eggs, including *H. halys, M. sexta*, and *P. xylostella*. Our results indicated that the MB had potent ovicidal effects with an LC_50_ value at 0.020 mg/cm^2^ and LC_95_ value at 0.048 mg/cm^2^ on *H. halys* (Table 3). A lower concentration of MB (0.0637 mg/cm^2^ active ingredient) was needed to reach 100% egg mortality for *H. halys* comparing to the other products used in the study (Table 4). At 0.0318 mg/cm^2^, MB was as potent as deltamethrin (0.0019 mg/cm^2^), *ζ*-cypermethrin (0.0223 mg/cm^2^), carbaryl (0.0080 mg/cm^2^), pyrethroid (*β*-cyfluthrin, 0.1592 mg/cm^2^), sulfur/pyrethroid (sulfur/pyrethrin, 0.6525 mg/cm^2^), and one of the organic essential oil products (2-phenethyl propionate, clover oil, rosemary oil, and thyme oil, at 0.3979 mg/cm^2^). Commercially available pesticides, *λ*-cyhalothrin (0.0016 mg/cm^2^), and acetamiprid, (0.0004 mg/cm^2^) and another organic essential oil product tested containing rosemary oil and peppermint oil (0.0637 mg/cm^2^) were almost ineffective. MB not only exhibited excellent ovicidal toxicity, but also had contact nymphicidal effect against *H. halys* nymphs (Table 1).

The MB was also ovicidal against *M. sexta* eggs at 0.0637 mg/cm^2^ dose with an LC_50_ value at 0.015 mg/cm^2^ and LC_95_ value at 0.060 mg/cm^2^ (Table 3). It was significantly better than the mixture of bifenthrin & *ζ*-cypermethrin (0.0239 mg/cm^2^) and an essential oil product containing 2-phenethyl propionate, clover oil, rosemary oil, and thyme oil (0.3979 mg/cm^2^) (Table 4).

For *P. xylostella*, MB demonstrated potent ovicidal activity at a dose as low as 0.0032 mg/cm^2^, with an LC_50_ value at 0.001 mg/cm^2^ and LC_95_ value at 0.005 mg/cm^2^ (Table 3). Interestingly, the carbaryl was one of the most effective compounds against *H. halys* egg at 0.0080 mg/cm^2^, but it was one of the most ineffective ovicidal compounds against *P. xylostella* (Table 4).

## Discussions

This current study demonstrates that methyl benzoate (MB) is an efficient green pesticide against invasive insect pest *D. suzukii,* and several other agricultural pests. MB not only effectively prevented *D. suzukii* from oviposition and inhibited subsequent larvae/pupae development, but also caused complete mortality of adult flies on pre- and post-treated blueberries at a concentration as low as 1%. Moreover, MB possessed ovicidal activity against several different species of eggs, when compared to some commercially available pesticides. On the basis of toxicity data, MB was five times more toxic than the conventional pyrethroid (*β*-cyfluthrin), 20 times more toxic than sulfur & pyrethrin mixture, and 12 times more toxic than one of the organic commercial products (2-phenethyl propionate, clover oil, rosemary oil, and thyme oil) against *H. halys* eggs. Neither *γ*-cyhalothrin nor acetamiprid exhibited ovicidal toxicity against *H. halys* at tested doses. For *M. sexta* and *P. xylostella*, similar toxic results were obtained, but *P. xylostella* appeared to be more sensitive to MB treatment. To reach 100% egg mortality, only 0.0064 mg/cm^2^ was needed, which was 10 times less than *H. halys* and 20 times less than *M. sexta* eggs needed for the same results.

In nature, many plant species emit a great amount of VOCs into atmosphere, which are related to plant ecology, physiology, and atmospheric chemistry^16^,^17^. Some of these VOCs may act as defensive compounds against insect herbivores and plant pathogens; while others may act as chemical signals involved in plant-plant, plant-animal, and plant-microorganisms interactions^18^. MB naturally occurs as an aroma and scent of many plants^19^, including flowers^20^,^21^ and fruits^22^,^23^,^24^,^25^,^26^,^27^, and plays important roles in plant communication with the surrounding environment. Particularly, MB is one of the more abundant scents emitted from petunia, *Petunia hybrid* and snapdragon, *Antirrhinum majus*, functioning as a long-range attractant to lure bees for pollination^28^,^29^,^30^,^31^. MB has also been used by many insect species as a semiochemical that carries a message for purpose of communication between individuals of the same species (intraspecific) or between different species (interspecific)^32^. Moreover, MB is known for its sweet, balsamic, spicy, and heady floral odor; and it has been used as a fragrance ingredient and preservative in many personal care applications, such as shampoos, shower products and face/neck care, liquid soaps, mouthwash, perfume, hair colorants and cosmetics^33^. MB is of low to moderate human toxicity by ingestion and inhalation^34^,^35^, and it is approved by the US Food and Drug Administration (21 CFR 172.515)^36^ and the European Union (EU Regulation 1334/2008 & 178/2002)^37^ for food use as a food-grade flavor ingredients. While MB is also considered environment-friendly, slowly biodegrading in the atmosphere^38^, it would still need to be registered as a pesticide with the EPA. To the best of our knowledge, the pesticidal activity of MB has not been previously reported. Overall, our research findings demonstrated that the methyl benzoate was an effective green pesticide against some invasive species, especially, *H. halys* and *D. suzukii*, with low concentration and high mortality; therefore, providing an alternative tool to synthetic pesticides for insect pest management in crop production.

## Methods

### Chemicals

Methyl benzoate, *α*-terpinene, *γ*-terpinene, terpineol, cineole, *α*-pinene, Tween 20, and Tween 80 were purchased from Sigma-Aldrich (St. Louis, MO). Acetone was used as solvent and purchased from Sigma-Aldrich (St. Louis, MO). All chemicals were used without further purification. Commercial pesticides were purchased from the Home Depot (College Park, MD) and used directly: Spectracide^®^ Bug Stop (St. Louis, MO), Bayer Advanced^®^ Carpenter Ant & Termite Killer Plus (Research Triangle Park, NC), Hot Shot^®^ Bedbug & Flea Home Insect Killer (St. Louis, MO), Raid Max^®^ Bug Barrier (Racine, WI), Amdro Quick Kill^®^ Lawn & Landscape Insect Killer (Atlanta, GA), Ortho^®^ Bug B Gon (Marysville, OH), Natria^®^ Insect Disease & Mite Control (Research Triangle Park, NC), Bayer Advanced^®^ Complete Insect Killer (Research Triangle Park, NC), Ortho^®^ Flower, Fruit & Vegetable Insect Killer (Marysville, OH), Sevin^®^ Garden Tech(Atlanta, GA), EcoSmart^®^ Organic Home Pest Control (Roswell, GA), and EcoSmart^®^ Organic Garden Insect Killer (Roswell, GA). Some commercial pesticides contained either a synthetic pyrethroid, a neonicotinoid or a combination of these. One (Sevin) contained a carbamate, while several other contained compounds (essential oils; and 2-phenylethy proprionate) exempt from EPA registration (see: https://www.epa.gov/minimum-risk-pesticides/minimum-risk-pesticide-definition-and-product-confirmation). Active ingredients and corresponding concentrations are listed in Table 2.

### Insects

The *H. halys* adults, nymphs, and eggs were obtained from colony maintained in the facility located in USDA, ARS, Beltsville, MD. The *H. halys* colony was established in 2007 from adults collected in Allentown, PA, USA. Insects were reared on a diet of organic green beans and shelled sunflower and buckwheat seeds (2:1, w/w) in ventilated plastic cylinders (21 cm × 21 cm OD, BioQuip Inc, Rancho Dominguez, CA) and maintained in Percival incubator (Percival Scientific Inc, Perry, IA) at 25 °C and 60% RH, under a 16 L:8D photoperiod^39^. Eggs were collected weekly and hatched in plastic Petri dishes with a water vial, and after molting to second-instars, the nymphs were transferred to the ventilated plastic cylinders for the remaining four instars^39^. Adult males and females were separated 1 or 2 days after emergence and subsequently maintained in different containers.

The colony of *M. sexta* was originally obtained from the University of Arizona, Tucson, AZ, reared, and maintained on an artificial wheat germ diet^40^ in an insectary located in the same USDA, Beltsville facility at 24 °C and 40% RH. Eggs and young larvae were covered by glass trays. Older larvae were kept in ventilated plastic boxes (27 × 17.5 × 10 cm, BioQuip Inc, Rancho Dominguez, CA). Adults were kept in screened cages (45.75 × 45.75 × 45.75 cm, BioQuip). After small tomato plants introduced into the screened cages for 3–4 days, deposited eggs on the plants were removed by hand.

The *P. xylostella* colony was obtained from Benzon Research, Carlisle, PA, reared, and maintained on an artificial wheat germ diet^41^ at the same USDA facility. Eggs and larvae were put in closed cardboard cups (236 mL, 8.9 cm diameter, 5.7 cm height, Solo Cup Company, Lake Forest, IL) and kept in a Percival incubator at 25 °C, 34% RH, under a 16 L:8 D photoperiod in the same insectary. Adults were maintained in screened cage (30.5 cm × 30.5 cm × 30.5 cm, BioQuip Inc). Eggs were deposited on aluminum foil strips (approx. 5.0 × 30.5 cm) dipped in cabbage juice and collected after 3–4 days.

The *D. suzukii* colony was provided by Rutgers University, originally obtained from *D. suzukii*-infested blueberry (*Vaccininum corymbosum* cv. Bluecrop) fruits in Burlington County, New Jersey. The colony was reared on cornmeal diet^42^ in polystyrene vials (height, 95 mm, diameter, 28.5 mm, Fisher Scientific, PA, USA) with plugs (height, 25 mm, diameter. 28.5 mm, Fisher Scientific, PA, USA) and kept in a Percival incubator at 25 °C, 34% RH, under a 16 L:8 D photoperiod in the same facility located in USDA, ARS, Beltsville, MD.

Organic green beans and blueberries (Cottle Farms, Cottle Strawberry Nursery, Inc, Faison, NC) were purchased from MOM’s organic market (College Park, MD, USA).

### Laboratory bioassay

Bioassays were conducted in a USDA Beltsville laboratory at ~25 °C controlled by central air-condition system, ~60% RH by a dehumidifier (WHYnter the Home Depot, MD), under a 16 L:8D photoperiod (TORK 1101 Time Switch, Amazon.com) with ∼1700 lux light illuminance (a 100 watt bulb). A fume hood was maintained at same condition with face velocity at 129 FPM. The plastic cups (32 oz, diameter 4.5 inches, deep 5 inches, or 16 oz, diameter 4.5 inches, deep 2.5 inches) were purchased from papermart.com (CA). An 80 mm diameter hole was cut from each lid and replaced with an 85 mm diameter mesh (mesh size, 81 × 81, BioQuip, CA) screen that was glued into place. The polystyrene vials (height, 95 mm, diameter, 28.5 mm) and plugs were obtained from Fisher Scientific (Pittsburg, PA). The plastic cage (30 × 30 × 30 cm) was purchased from BugDorm (Rancho Dominguez, CA). Glass vial (20 mL), glass spray bottle (Amber glass with spray top, 30 mL), Petri dish (9 cm diameter), and Whatman filter paper (90 mm diameter) were obtained from VWR (Atlanta, GA). Deionized water (DI) containing 1% emulsifier (v/v), Tween 20 and Tween 80, at 1:1 ratio was used to make different VOCs water solutions and also used as blank control.

### Toxicity of MB to *D. suzukii*

To investigate the acute toxicity of MB against *D. suzukii*, the bioassays were performed according to a published procedure^43^ with modifications. A total of 100 blueberries were placed in a plastic cage (30 × 30 × 30 cm) and infested by 100 mixed sex adults *D. suzukii* for 4 days. After removing all insects, half of pre-infested blueberries were dipped in100 mL MB aqueous emulsion at 1% concentration, while other half of pre-infested blueberries was dipped in deionized water (DI) water as a control. The blueberries were subsequently placed in two separate Petri dishes and allowed to air dry for 2 hr. After drying, the blueberries were stored separately in two plastic cups (32 oz) with ventilated lids and incubated at ambient room temperature on the bench top for 12 days. At this time, the presences of adult *D. suzukii* were recorded, and the berries were dissected to assess development of any larvae and pupae. The experiment was repeated six times.

### Comparison of MB to ‘minimum risk’ pesticides against *D. suzukii*

To compare the pesticidal properties of MB to other essential oils (considered ‘minimum risk pesticides’) against *D. suzukii*, a series of bioassays were conducted according to the published procedure^43^ with modifications. 10 blueberries were separately dipped in either 100 mL MB, or other essential oil aqueous emulsions (1%) Blueberries dipped in 100 mL DI water served as controls. The blueberries were placed in different Petri dish bottoms and allowed to air dry for 2 hr. After drying, the treated blueberries were placed in plastic cups (32 oz) with ventilated lids, and 10 adult (males and females) *D. suzukii* were introduced into each cup. Mortality of the *D. suzukii* was examined after 48 hr. After removing all insects, the blueberries were maintained at room temperature on the bench top for another 10 days. Present of adults were subsequently assessed and development of larvae and pupae was further inspected by dissection of the berries.

### Toxicity of MB to *H. halys* nymphs

The contact mortality bioassays were carried out in scintillation glass vials (20 mL, Wheaton Scientific Product, Millville, NJ), following published procedures with modifications^44^. Filter paper was cut into round pieces (2.4 cm diameter). MB was added to acetone to give different concentrations (0.025, 0.05, 0.10, 0.25, 0.5, 1.0, 2.0, 4.0%). 50 μL of each solution was applied to the filter papers, and the filter papers were dried for 1 min before being placed in the bottom of the vials. A small piece of green bean (1 cm) was put on the filter paper in each vial as food source. Different stages of *H. halys* nymphs were introduced into the vials and then the vials were capped with loosen cotton balls to prevent the nymphs from escaping. For each stage, 30 nymphs were used for each concentration of MB and nine different concentration solutions (including blank control) were tested. In total, 270 nymphs were tested for each stage. Due to the size and weight of *H. halys* increased significantly from first to fifth instar, the number of nymph tested per vial was decreased from 10 to 2 accordingly so that they had enough space and could move freely in the vial. For example, for the first instar, 10 nymphs were tested pre vial (3 vials per concentration); for the second and third instars, 5 nymphs were tested per vial (6 vials per concentration); for the fourth instar, 3 nymphs were tested pre vial (10 vials per concentration); and for the fifth instar, 2 nymphs were tested pre vial (15 vials per concentration). The vials were maintained in a fume hood and mortality was assessed after 24 hr. Mortality data was subjected to probit analysis using PoloPlus for LC_50_, LC_95_ with 95% confidence intervals calculation. In all tests acetone acted as the control.

### Ovicidal toxicity of MB and commercial pesticides

Aqueous solutions of MB at varying concentrations (0.00, 0.025, 0.05, 0.10, 0.25, 0.5, 1.0, 2.0, 4.0%) and commercially available pesticides (see Table 2) were tested against several insect species. Eggs (10 for *H. halys* and *M. sexta*, 100 for *P. xylostella*) were collected and then placed on filter paper in Petri dishes. Different aqueous solutions were sprayed on the surfaces of the eggs three times (~ 0.5 mL total) using glass spray bottles (Amber glass with spray top, 30 mL) to completely cover the treatment areas (egg and filter paper). Then Petri dishes were covered with lids and maintained in a fume hood for 10 days. After this time, the Petri dishes were then inspected for presence of nymph or larvae, and the number of unhatched eggs, if any. An acetone spray was used as a control. The experiment was repeated three times.

### Data analysis

Comparisons of different treatments were analyzed using one-way ANOVA followed by Ryan-Einot-Gabriel-Welsch F test (SPSS 10.0 for Windows)^45^ for significance at α = 0.05). Some data that ranges from being heavily positively skewed distribution (skewness = 1.37–2.38); therefore, log transformations were performed to remedy non-normality prior to the statistical analysis. PoloPlus software (LeOra Software, Berkeley, CA) was used to conduct probit analysis for mortality data, and LC_50_ and LC_95_ with 95% confidence intervals were estimated.

## Additional Information

**How to cite this article:** Feng, Y. and Zhang, A. A Floral Fragrance, Methyl Benzoate, is An Efficient Green Pesticide. *Sci. Rep.* **7**, 42168; doi: 10.1038/srep42168 (2017).

**Publisher's note:** Springer Nature remains neutral with regard to jurisdictional claims in published maps and institutional affiliations.

## Figures and Tables

**Table 1:** Nymphicidal toxicity of MB to different stages of *H. halys.*

Stage	n*	LC_50_ (95% CL) μL/vial	Slope ± SE
1^st^	270	1.03 (0.93–1.10)	7.69 ± 1.07
2^nd^	270	1.01 (0.86–1.12)	6.73 ± 1.11
3^rd^	270	1.23 (1.12–1.33)	5.28 ± 0.60
4^th^	270	2.39 (2.19–2.60)	6.10 ± 0.72
5^th^	270	1.77 (1.60–1.93)	6.00 ± 0.67

^*^Number of nymphs tested, 30 nymphs/concentration and nine different concentration solutions (including blank control) were tested. Acetone was used as blank control.

**Table 2:** Commercially available pesticides tested in laboratory bioassay.

Trademark	Product	Active Ingredient (AI)	C%*
Spectracide	Bug Stop	Gamma-cyhalothrin	0.025
Bayer Advanced	Carpenter Ant & Termite Killer Plus	Beta-cyfluthrin	2.5
Hot Shot	Bedbug & Flea Home Insect Killer	Lambda-cyhalothrin	0.03
Raid Max	Bug Barrier	Deltamethrin	0.03
Amdro Quick Kill	Lawn & Landscape Insect Killer	Zeta-cypermethrin	0.35
Ortho	Bug B Gon	Bifenthrin	0.3
		Zeta-cypermethrin	0.075
Natria	Insect, Disease & Mite Control	Sulfur	10
		Pyrethrin	0.25
Bayer Advanced	Complete Insect Killer	Imidacloprid	0.72
		Beta-cyfluthrin	0.36
Ortho	Flower, Fruit & Vegetable Insect Killer	Acetamiprid	0.006
Sevin	GardenTech	Carbaryl	0.126
EcoSmart	Organic Home Pest Control	2-phenethyl propionate	5
		Clover oil	0.5
		Rosemary oil	0.5
		Thyme oil	0.25
EcoSmart	Organic Garden Insect Killer	Rosemary oil	0.5
		Peppermint oil	0.5

^*^Aqueous solution.

**Table 3:** Ovicidal toxicity of MB to three species of insect eggs.

Insect	n*	LC_50_ (95% CL) mg/cm^2^	LC_95_ (95% CL) mg/cm^2^	Slope ± SE
*H. halys*	270	0.020 (0.012–0.026)	0.048 (0.036–0.090)	4.36 ± 1.11
*M. sexta*	270	0.015 (0.011–0.020)	0.060 (0.042–0.112)	2.77 ± 0.46
*P. xylostella*	2100	0.001 (0.001–0.002)	0.005 (0.004–0.025)	7.32 ± 1.14

^*^Number of eggs tested. 30 eggs/concentration and nine different concentration solutions (including blank control) were tested for H. halys and M. sexta. 300 eggs/concentration and seven different concentration solutions (including blank control) were tested for P. xylostella. DI water solution contained 0.5% Tween 20 (v/v) and 0.5% Tween 80 (v/v) was used as blank control.

**Table 4:** Ovicidal effect of MB and tested commercially available pesticides on different species of eggs after 10 days exposure.

Treatment*	AI Dose (mg/cm^2^)	Hatchability (%) (mean ± SE)		
		H. halys**	M. sexta**	P. xylostella***
Blank Control****	0.0000	70 ± 5.8^*d*^	87 ± 8.8^*c*^	78 ± 3.8^*d*^
MB 0.025%****	0.0016	67 ± 8.8^*d*^	67 ± 8.8^*c*^	38 ± 5.5^*c*^
MB 0.05%****	0.0032	63 ± 17.6 ^*cd*^	63 ± 8.8^*c*^	13 ± 5.0^*ab*^
MB 0.1% ****	0.0064	60 ± 10.0 ^*cd*^	50 ± 17.3^*c*^	0 ± 0.3^*a*^
MB 0.25 ****	0.0159	43 ± 6.7^*abcd*^	40 ± 15.3^*c*^	1 ± 0.0^*a*^
MB 0.5% ****	0.0318	17 ± 12.0^*ab*^	20 ± 10.0^*abc*^	0 ± 0.0^*a*^
MB 1% ****	0.0637	0 ± 0.0^*a*^	3 ± 3.3^*ab*^	0 ± 0.0^*a*^
MB 2% ****	0.1273	0 ± 0.0^*a*^	0 ± 0.0^*a*^	
MB 4% ****	0.2546	0 ± 0.0^*a*^	0 ± 0.0^*a*^	
Bug Stop (*γ*-cyhalothrin)	0.0016	83 ± 8.8^*d*^	0 ± 0.0^*a*^	9 ± 5.4^*a*^
Carpenter Ant & Termite Killer Plus (*β*-cyfluthrin)	0.1592	3 ± 3.3^*a*^	0 ± 0.0^*a*^	7 ± 1.2^*a*^
Bedbug & Flea Home Insect Killer (*λ*-Cyhalothrin)	0.0019	53 ± 3.3^*bcd*^	0 ± 0.0^*a*^	2 ± 0.9^*a*^
Bug Barrier (Deltamethrin)	0.0019	10 ± 5.8^*ab*^	0 ± 0.0^*a*^	3 ± 1.0^*a*^
Lawn & Landscape Insect Killer (*ζ*-cypermethrin)	0.0223	0 ± 0.0^*a*^	0 ± 0.0^*a*^	1 ± 0.9^*a*^
Bug B Gon (Bifenthrin & *ζ*-cypermethrin)	0.0239	33 ± 14.5^*abcd*^	43 ± 14.5^*c*^	1 ± 0.7^*a*^
Insect, Disease & Mite Control (Sulfur & Pyrethrin)	0.6525	3 ± 3.3^*a*^		
Complete Insect Killer (Imidacloprid & *β*-cyfluthrin)	0.0688	0 ± 0.0^*a*^		
Flower, Fruit & Vegetable Insect Killer (Acetamiprid)	0.0004	67 ± 20.3^*d*^		16 ± 7.0^*ab*^
GardenTech (Carbaryl)	0.0080	0 ± 0.0^*a*^		28 ± 9.2^*bc*^
Organic Home Pest Control				
(2-phenethyl propionate & clover oil & Rosemary oil & Thyme oil)	0.3979	17 ± 3.3^*ab*^	30 ± 17.3^*bc*^	1 ± 0.3^*a*^
Organic Garden Insect Killer (Rosemary Oil & Peppermint Oil)	0.0637	70 ± 25.2^*d*^		

^*^0.5 ml volume applied. ^**^ 30 eggs per treatment. ^***^ 300 eggs per treatment. ^****^ DI water solution contained 0.5% Tween 20 (v/v) and 0.5% Tween 80 (v/v). Means within the same column flowed by the different letters are significantly different at α = 0.05 (Log transformed, *N* = 3; for H. halys, df = 20, 42, F = 9.41, p < 0.001; for M. sexta, df = 15, 32, F = 14.14, p < 0.001; for P. xylostella, df = 15, 32, F = 29.18, p < 0.001).
